# Intimate partner violence against women and its related immigration stressors in Pakistani immigrant families in Germany

**DOI:** 10.1186/2193-1801-1-5

**Published:** 2012-06-21

**Authors:** Rubeena Zakar, Muhammad Z Zakar, Thomas Faist, Alexander Kraemer

**Affiliations:** 1 Department of Public Health Medicine, School of Public Health, Bielefeld University, Universität Straße 25, Bielefeld, 33615, Germany; 2 Institute of Social and Cultural Studies, University of the Punjab, Quid-e-Azam Campus, Lahore, 54590, Pakistan; 3 Centre on Migration, Citizenship and Development (COMCAD), Faculty of Sociology, Bielefeld University, Universität Straße 25, Bielefeld, 33615, Germany

**Keywords:** Intimate partner violence, Immigrant families, Pakistan, Immigration stressors

## Abstract

This paper addresses the issue of intimate partner violence against women and its related immigration stressors in Pakistani immigrant families in Germany. Drawing on 32 in-depth interviews with Pakistani women in three cities in Germany, we found that psychological violence was the commonly reported violence among the study participants. The data showed that the process of immigration exacerbated tensions between spouses because of various immigration stressors such as threats to cultural identity, children’s socialization, and social isolation. In order to cope with the stressful spousal relations, women applied various indigenous strategies, but avoided seeking help from the host country’s formal care-providing institutions. This study also debunks some stereotypes and popular media clichés about the “victimhood of women from conservative developing countries” and provides an understanding of the issue of intimate partner violence within an immigration context. Further research with a larger sample will be helpful to understand immigration-induced stress and intimate partner violence in immigrant families.

## Background

Intimate partner violence (IPV) against women is a serious human rights problem world-wide (United Nations Fourth World Conference on Women [1995]). Despite differences in culture, religion, and customs, IPV occurs in both developed and developing countries. IPV is also reported in immigrant, minority, and marginalized communities. However, the impact of migration on IPV has not yet been comprehensively investigated, though recently some studies have addressed this issue (e.g. Menjivar and Salcido [2002]).

The nationally representative data from any host country on the prevalence of IPV in immigrant families is unavailable (Menjivar and Salcido [2002]). Nonetheless, various small sampled studies have reported that immigrant women from Asian and African countries in a range of host settings frequently experience IPV (Raj and Silverman [2002a]; Leung and Cheung [2008]; Sullivan et al. [2005]; Thapa-Oli et al. [2009]; Ahmad et al. [2004]). Prior research has also suggested that sometimes IPV is tied to immigration-related stressors like discrimination and racism, language barrier, clashing cultural values, and social isolation (Abraham [1998]; Dasgupta [2000]; Raj et al. [2005]).

### Cultural context of IPV in Pakistani immigrant families

IPV is a complex and multifaceted phenomenon. In popular discourse, IPV is projected as random, routine or a normal emotional occurrence between husband and wife. Nonetheless, recent research has shown that various individual, community and socio-cultural factors (Heise [1998]) provide a context wherein spousal power relations defined; and violence is used as a tool by the husband to maintain the asymmetry of relations and to ensure the dominance and control over wife.

Like other South Asian women, some Pakistani women may also be the victims of IPV when they migrate to other countries. It is argued that in Pakistan gender relations are based upon structures of oppression that are deeply embedded in its distinct geography, history, and culture (Critelle and Willett [2010]). When Pakistani families migrate to other countries, they may try to retain their values and norms. As is the case in their home country, husbands may continue to expect a subservient role on the part of their wives (Abraham [1998]).

Usually, in Pakistan, women are brought up and socialized according to patriarchal norms (Ayyub [2007]). From the early stages of socialization, girls are taught obedience and submission to their male guardians (Abraham [1998]; Ahmad et al. [2004]; Raj and Silverman [2002b]). Normatively, mothers provide training for their daughters about their culturally expected gender-roles. Usually, daughters also learn “appropriate” gender-roles by watching their “mothers-fathers” relations. Culturally, a girl learns that the primary focus of her life should be the development, care, and service of her family (Ayyub [2000]). Any conflict or dispute within the family is considered a sign of her incompetence. Within this scheme of things, an ideal wife must sacrifice her personal desires, minimize conflicts, hide problems and bear suffering for the sake of her husband and children (Abraham [1998]). Overall, she is trained to perform passive, dependent, and subordinate roles (Ayyub [2000]).

Nonetheless, Pakistani society is not homogenous in terms of gender roles: there is religious (rigid versus liberal interpretation), regional (underdeveloped versus prosperous), geography (rural versus urban), ethnic, and class (uneducated working class versus educated white collars) diversity. For example, restrictive and passive gender roles may more likely to be transmitted and adhered to by the families having low level of education and conservative orientations.

Despite these social class differentials in gender-roles, the overall personality of a girl is expected to be a “bit shy and submissive”. If she poses herself as “too independent” or “individualistic,” her parents may become worried about her future. Culturally, such behavior may be problematic for her marital life. To formulate her behavior to be consistent with gender-role expectations, she is constantly reminded by her family and relatives to behave in a “proper, submissive, and respec’ manner.” Any deviance from traditional gender-roles may bring shame and dishonor to the family (Abraham [1998]; Ho [1990]). After marriage, she is supposed to submit and obey her husband. Culturally, a husband has the right to monitor and control the behavior and conduct of his wife, and in the case of inappropriate behavior on her part, he is entitled to “punish” her (Ayyub [2000]; Busby [1999]).

Once married, the message is ingrained in a woman by her parents, friends, and clergy that the marriage should be maintained at any cost (Ayyub [2007]). If she gets divorced, she will not only bring shame on the family but also a devastating stigma upon herself (Shirwadkar [2004]). Therefore, some women, even in the face of persistent IPV, continue to stay in the abusive relationship (Bui [2004]; Ho [1990]; Critelle and Willett [2010]). In some situations, in this cultural context, a husband considers it his prerogative to control and correct the behavior of his wife (Dobash and Dobash [1979]) and if the need arises he can “discipline” her by using physical punishment (Huisman [1996]; Kim et al. [2007]; Kulwicki and Miller [1999]). In such an environment, IPV is tolerated, sustained, and perpetuated (Ayyub [2007]; Kulwicki and Miller [1999]) as well as socially endorsed (Bhuyan and Senturia [2005]).

### Immigration context

The process of immigration exposes people to totally different socio-cultural and economic systems. Almost all types of immigrants face some difficulties in settlement and adjustment process, but the undocumented immigrants or those who are less educated, poorly trained or lacked proper competencies to understand the host country’s social or economic system may likely to face more difficulties and settlement challenges in the host country (Samuel [2009]). Understandably, in the new set-up, they may encounter various stressful situations because of language barriers, limited economic resources, discrimination and racism, clashing cultural values, and social isolation (Bui [2003]; Kim et al. [2007]). As a result, immigrants often experience a deep sense of loneliness in the new environment (Kang and Kang [1983]). This loneliness and powerlessness is further exacerbated by immigrants’ lack of social competence and absence of cultural capital to become integrated within the host society (Hughes and Gove [1981]).

Cumulatively, all these factors create a stressful situation for immigrant families, which may in turn increase the families’ vulnerability to tense and strained interpersonal relations (Erez [2000]; Gelles [1985]; Straus [1990]). In stressful and difficult situations, wives, being relatively less powerful and more dependent on their husbands, are more likely to be the victims of their husbands’ anger (Ahmad et al. [2004]; Sullivan et al. [2005]). Research has reported that a higher degree of stress experienced by a husband is positively associated with a greater likelihood of abusing his wife (Kim and Sung [2000]).

### Pakistani immigrant families in a western culture

In Pakistani culture, the power position of family members is rigidly defined by variables such as age, gender, and economic contributions (Ayyub [2000]). Members of the extended family are expected to have faith in the head of the family, who is usually a father or husband (Ayyub [2000]). When these families migrate to Western industrialized countries, the expectation is that the family should retain its hierarchal, gender-based power structure.

Life in Western countries seems very attractive when immigrant families plan to migrate. But when they encounter the reality of the “free countries,” men may not necessarily like it. As Ayyub ([2000]) reported: “Men accustomed to a patriarchal family system now found it difficult to share power with their wives” (p. 244). Despite the efforts of men to minimize the impact of the host society on their women, they cannot stop the massive and all-encompassing power of the culture to influence individuals.

The new social environment may create irritants and tensions between immigrant couples (Dion and Kawakami [1996]). These tensions may not be exclusively based on a dominance-subordination struggle between husband and wife but also on serious disagreements about the socialization of children, especially adolescent daughters (Mehrotra and Calasanti [2010]). As the children grow, their parents’ concern about the impact of Western culture on them also grows. Normally, the socialization of children is the primary responsibility of the mother (Mehrotra and Calasanti [2010]); any failure in this context can result in tension between husband and wife. For example, if a young daughter commits any acts of “moral deviance”, the mother could be blamed for her failure to socialize her daughter.

There are other migration stressors that could cause tension and conflict between husband and wife (Menjivar and Salcido [2002]; Ilkkaracan [1996]). Research reports that, in host societies, the extended family may sometimes also play a role that exacerbates tensions between husband and wife and could result in IPV (Dasgupta [2000]; Mehrotra [1999]; Lee [2000]). However, other studies yield the contradictory finding that interaction with an extended family provides a buffer against partner violence, as the extended family provides social support, financial resources, child-care and protection from violence (e.g. Kasturirangan et al. [2004]; Sharma [2001]). Overall, culturally constructed gender identities and rigid gender roles and stereotypes have been facilitating the abuse of women in immigrant populations (Bui and Morash [1999]; Tran and Des Jardins [2000]). However, when Pakistani families migrate to other countries, the institutional structures and normative conditions change; and the migrating couple faces a new social environment.

### Pakistani immigrant families in Germany

It is estimated that the total Pakistani immigrant population in Germany is about 70,000 and most of them are settled in big cities like Frankfurt, Berlin, and Hamburg (Pakistani Consulate Frankfurt 2011, personal communication). Historically, most of Pakistanis immigrant population is settled in the English speaking countries like USA, England and Australia. However, in the late seventies, Pakistani government officially declared *Ahmadis* as non-Muslims and Ahmadis claimed that Pakistani state laws are discriminatory against them (Human Right Watch [2010]). As a result, a substantial number of Ahmadi community sought political asylum in Germany. Presently, the estimated number of Pakistani Ahmadi community in Germany is 25,000 (personal communication, second author). In addition to these political refugees, a substantial number of unskilled working class entered in Germany as political asylum seeker. However in the early 2000s, some qualified professionals such as information technology experts and students arrived in Germany for white collar jobs and higher education. Presently, in Germany, Pakistani community consisted of unskilled workers (65%), semi-skilled workers and small-scale business owners (20%), white collar workers (5%), students (5%), and undocumented migrants (5%) (personal communication).

Thousands of Pakistani families have migrated to Germany and many thousands more aspire to migrate in the future (using matrimonial connections). Thus far, to the best of researchers’ knowledge, no scientific study has been conducted to understand the dynamics of IPV in Pakistani immigrant families in Germany. The aim of this study was to examine the Pakistani immigrant women’s perceptions about and experiences of IPV especially after migration to Germany and how various immigration stressors could influence familial relations in the host country.

## Methods

The study was based on 32 in-depth interviews conducted in three relatively small cities that are Bielefeld, Herford and Osnabruck, situated in the North of Germany. The in-depth interviews were conducted with married Pakistani immigrant women of reproductive age who were married to Pakistani men and came from Pakistan with their husbands or after marriage sponsored by them.

### Sample size and recruitment of respondents

A snowball sampling technique was used to recruit the women for in-depth interviews. For the recruitment of Pakistani women, the members of local community organizations working for South Asian immigrants were contacted. They explained the study objectives to women of Pakistani origin during different cultural events and gatherings. Initially, three women expressed their willingness to participate in the study. These three interviews took place at office of the community organization in a separate room. At the end of each interview, these three women were asked to refer other women in their social network for participation in the study (Henry [1990]). This procedure was repeated with each new participant until the researcher reached saturation point, finally, 32 in-depth interviews were completed (see Table 1). Neither the woman who identified the other women nor the researcher had any prior knowledge of whether the participants who were recruited for the study had any experience of IPV. The study was approved by the relevant institutional ethical committee.

**Table 1:** Total population of three cities, number of Pakistani immigrants, and drawn sample

**Name of cities**	**Total population**^**a**^	**Total number of Pakistani immigrants**^**b**^		**Sample drawn**
		**Men**	**Women**	
Bielefeld	302,300	137	80	17
Osnabruck	163,357	93	71	9
Herford	55,700	62	36	6
Total	521,357	292	187	32

a *Statische Bundesamt Deutschland* 2010.

*b* Statistics gathered from *Bundesamt für Migration und Flüchtlinge* (Migrants and refugee office) by personal communication.

Interviews were semi-structured; a checklist of specific topics was used for conducting in-depth interviews with the participants. The checklist was developed on the basis of a review of the literature and two informal discussions with Pakistani immigrant women on the topic. The in-depth interviews began with questions regarding the socio-economic characteristics of the women and gradually moved from broad questions on life in Germany, differences between Pakistani and German society, and migration related stressors. The questions then narrowed to their perceptions of and attitudes to IPV, their experiences of psychological and physical violence (because of cultural sensitivities, any discussion of sexual violence was avoided) from their husbands. All questions were worded open-ended. Questions related to women’s life after migration in Germany and their marital experiences were asked in following ways: “What type of changes did you feel after coming to Germany? What type of stressful situations did you experience after coming to Germany? Tell me about your experiences of living with your husband after coming to Germany? What kinds of actions did you take to cope with your relationship related tense situations?” Women were encouraged to express their views frankly and openly.

### Interview process

All the qualitative data were collected by the first author and one female researcher. Both interviewers being Pakistani, immigrant, and married women, they developed a good rapport with the participants. Most interviews were conducted in the first language of the respondents (and interviewers), i.e. Urdu, but some participants preferred the Punjabi language, so six interviews were conducted in Punjabi. The interviews were conducted at respondents’ residence after ensuring the privacy and at the time of their convenience. Before the start of the interview, written informed consent was taken from all the participants. They were also informed about the reason for their selection and the maintenance of confidentiality. For ensuring privacy no personal information like names and addresses were asked, instead specific codes were used to identify the respondents’ responses. The duration of interviews ranged from one and a half to two hours. With the permission of participants, all interviews were audio-recorded as well as written notes being taken during the interviews. The confidentiality and safety of the respondents were ensured throughout the data collection process. The participants were offered an information list of social services and resources provided by local service agencies.

### Data analysis

All audio-recorded in-depth interviews were transcribed verbatim into written form by the first author. Interviews were initially transcribed into Urdu and were subsequently translated into English. Data were analyzed by using general inductive approach (Thomas [2003]) and both deductive and inductive reasoning were applied for analyzing the data. Initial, transcript coding was performed independently by each author and then in joint sessions involving all the authors. First, all the authors reviewed each transcript line-by-line to familiarize themselves with the content and gained an understanding of the “themes” and other details in the text. Secondly, the researchers identified and defined categories or themes. General categories were derived from the research aims (i.e., the original themes used in the interview schedule) such as migration stressors, and specific categories (e.g., problems in socialization of children and immigration-induced social isolation, etc.) were derived from multiple reading of the transcript. Thirdly, we searched the data for coherence and different ideas about the same phenomenon and overlapping coding were examined. Lastly, we searched subtopics within each category including contradictory points of view and new insights. We selected appropriate quotes that convey the core theme or essence of a category and categories having similar meaning were combined under a super-ordinate category (Thomas [2003]).

To ensure accuracy and consistency, the researcher met regularly during the process of analysis of the data, first to articulate and then to refine the qualitative categories used in the coding process. In order to preserve the validity of the responses, the first author shared the initial write-up with other researchers. Improvements were made according to their suggestions. Finally, the results were discussed with all the study participants. A few of them provided some clarifications and comments, which were incorporated into the final text.

### Participants’ characteristics

The women’s ages varied from 22 to 48. All the women were currently married except one, who had become divorced three years before. The women were from different geographical locations in Pakistan. The majority (18) of the participants were from Punjab, 9 were from South, and 5 were from North of Pakistan. Most of the women were from the lower middle class. Three had no children, while the rest had one to four children, ranging in age from 2 to 17 years. The length of the women’s marriages varied from three to 22 years and the length of time between their marriages and their arrival in Germany varied from six months to six years. The women’s length of residence in Germany varied from two to 20 years (see Table 2).

**Table 2:** Socio-demographic characteristics of the respondents (N = 32)

**Characteristics**	
Age of women (years)	
Mean	31.00
Range	22-48
Age of husbands (years)	
Mean	36.00
Range	28-56
	n (%)
Familial take-home monthly income (in Euro)	
<1000	19 (59.4)
1000-1600	11 (34.4)
>1600	2 (06.2)
Education of women	
No schooling	4 (12.5)
Up to 10 years of schooling	21 (65.6)
> 10 years of schooling	7 (21.8)
Education of husbands	
No schooling	2 (6.2)
≤ 10 years of schooling	18 (56.3)
> 10 years of schooling	12 (37.5)
Participant employment status	
Housewife	30 (93.8)
Unskilled worker	1 (3.1)
Skilled worker	1 (3.1)
Husband’s employment status	
Unemployed	3 (9.4)
Unskilled & par-time workers	17 (53.1)
Skilled worker	8 (25.0)
Professional/managerial jobs	4 (12.5)
Religion	
Muslim	28 (87.5)
Muslim (Ahmadi)^a^	3 (9.4)
Christian	1 (3.2)
Number of children	
0	3 (9.4)
1-2	16 (50.0)
3-4	13 (40.6)
Marital status	
Currently married	31 (96.8)
Divorce	1 (3.1)
Length of residence in Germany (in years)	
<5	14 (43.7)
≥5	18 (56.3)

^a^ According to Constitution of Pakistan, Ahmadi sect is declared as non-Muslim. They claim that they are discriminated by Pakistani legal and social system.

## Findings

The focus of analysis of the qualitative data was to explore and understand the dynamics of IPV in Pakistani immigrant families in Germany as well as to examine the influence of the process of migration on spousal relations. Four main themes emerged from the analysis of data: (a) change of culture and familial relations; (b) Migration/acculturation stressors and abuse in spousal relations; (c) women’s experiences of IPV, and (d) women’s response to IPV.

### Change of culture and familial relations

Pakistan and Germany are markedly different societies in terms of historical traditions, religious orientations, behavioral norms, and family relations. Compared to Pakistan, Germany has attained a considerable degree of gender equality and women have a relatively high degree of participation in socio-economic life. Conversely, Pakistan is a conservative society where women, especially from low socio-economic and rural background, have a very low level of socio-economic participation and live under a rigid patriarchal structure (Critelli [2010]; Jafar [2005]).

Similarly, the process of migration from a developing country might be troublesome and difficult especially for those families have low level of education, restricted exposure to modernity and less pluralistic background (Rianon and Shelton [2003]). For example, in Pakistan, when people plan to migrate to Germany, they develop their own judgments and thoughts about the country of destination. Their judgments are based on stories from their friends and relatives, or information gained from TV and other forms of mass media. But when they actually enter the host country, they are surprised to find it a different world; very different from their imagination and expectations. When immigrants’ expectations are unmet, they can experience psychological crisis and social dysfunction, such as interpersonal stress, anger, sadness, and culture shock (Samuel [2009]).

We discussed these points with our study participants. During the course of the interviews, almost all the participants talked about cultural differences. The most striking difference they reported was the individualism and markedly different gender-roles in German society. While explaining the differences in gender-roles between Germany and Pakistan, one housewife in her late forties observed:

In Pakistan, we always think in terms of family. We work for our family members, we sacrifice for them. Here [in Germany] women are independent and self-centered. They earn for themselves, and take care of their individual self more than anything else. They have a good life; they go on holiday every six months. We cannot think of such a life in Pakistan. We have a different culture.

When immigrants from conservative societies experience various differences in the host country, they try to understand, interpret, and evaluate the impact of this changed environment on their lives. They try to learn and adjust to their new surroundings, but this process also creates some fear, ambivalence, and uncertainty. In their day-to-day life, they try to understand what is appropriate and inappropriate in the host culture. One high school educated housewife in her mid-forties, while narrating her experiences of immigration with special reference to the attitude of her husband, stated:

Here [in Germany] life is different; the nature of familial relations is different. Here people treat children differently and talk with their women politely. One day when my husband was talking with me in a shopping-mall [normal talking], people started staring at us. …my husband has a habit of speaking loudly. People thought that he was yelling at me.

Relocation from a conservative country, such as Pakistan, into a post modern country like Germany might pose a threat to immigrants’ self identity (Rianon and Shelton [2003]). During the discussion, we noted that some women felt a threat to their cultural identity: they wanted to preserve their core religious and family values. Nonetheless, they did not reject the host culture in total: they were willing to have selective assimilation with the economic mainstream of the host society.

During the interviews, women reported some “fears” that could create tension between husband and wife while being in Germany. A typical stereotype was the influence of Western culture on the morality of men. The availability of pornographic material was perceived by women as a threat to the “morality” of their families. One woman said that, when her husband departed for Germany, his father advised him “to refrain from immoral activities and to stick to the religious values.” One woman narrated the experience of her friend, who questioned her husband about his “immoral activities.” Such questions always create tension and anger between the husband and wife. Another woman, while narrating the experiences of her friend, opined: “In this relatively free and ‘secular’ society, some men tried to ‘benefit’ from the ‘freedom’, like drinking alcohol, watching pornographic films or having a girlfriend. But such ‘freedom’ was strictly forbidden for women.” She further observed: “There is much hypocrisy among some Pakistani men regarding their attitude towards women. Men have different moral standards for their wives and for themselves.”

In our study, no woman openly reported that her husband watches pornographic films or is involved in other “immoral activities”. However, two respondents hinted at some fear about the involvement of their husbands in such activities. So, in order to ward off such fears, individual and collective efforts were made to preserve their cultural identities and “moral purity”. One woman with a high school education highlighted the importance and centrality of Pakistani family values. She observed:

This free culture [Germany] could influence our women by giving them a sense of independence and liberty, which could ultimately weaken our value system, especially our family institutions. We therefore actively try to preserve our values and put up a collective action against a woman or a family who undermines our identity or destroys our value system.

### Migration/acculturation stressors and abuse in spousal relations

Adjustment and resettlement of Pakistani immigrant families to Germany is expected to be a challenging and difficult process. Research has reported that the process of immigration creates many unexpected stresses for the immigrants and among them one could be parent–child conflict which may be the result of dissonant acculturation ((Portes and Rumbaut [2001]). It happens when parents and children do not acculturate at similar pace, for example, when children acculturate faster or more completely than parents (Portes and Rumbaut [2001]). It may also happen when family members (e.g., husband and wife) perceive and incorporate the elements of host culture in different way and as a result their relations may experience strain and tense situations (Ahmad et al. [2004]; Samuel. [2009]). Different studies have conceptualized this phenomenon differently; some call it acculturation stress (Samuel [2009]) or migration stressors (Keygnaert and Temmerman [2007]).

**Figure 1:**
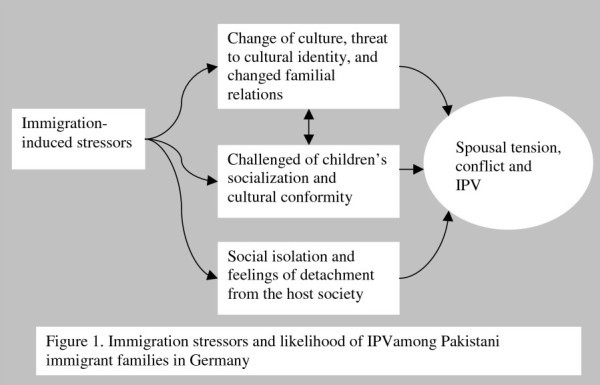
Immigration stressors and likelihood of IPVamong Pakistani immigrant families in Germany.

During the course of our interviews, the women explained their experiences of various stressors after arriving in Germany. The most commonly-experienced and most worrisome stressor was issues related to children’s socialization, and especially the concern to ensure the cultural conformity of adolescent daughters. Others stressors included the growing inter-generational gap, the threat of unemployment and loss of status, social isolation, and the unpredictable behavior of people in the host society (see Figure 1).

The socialization of children was the most important concern among the study participants. The women with daughters were more worried about their daughters’ socialization. Almost all the participants who had daughters believed that they should “keep an eye” on their daughters because they could not afford “too much freedom”, even though they pretended to be very liberal and Westernized. During the conversations, the women revealed that, although both husband and wife were responsible for the socialization of their children, the primary responsibility for proper socialization of children (according to their cultural values) rested with mothers. As per cultural values and gender roles, mothers were blamed and held responsible for any inadequate value inculcation and training of their children. One participant said:

*Taleemo-tarbiat* [cultural socialization] of children, especially daughters, is primarily the responsibility of the mother. If my daughter does not follow our culture and does not care about our cultural values then everyone will point the finger at me. …That I am not a good mother to teach my daughter about preserving the family honor and values. Here [in Germany], I am worried about how to save my daughter from *bayrahravi* [waywardness] and other evils of the society like free mixing of both genders.

Another widely expressed concern of the participants was the preservation of cultural identity in their children. We found that the issue of socialization of children was very sensitive between husband and wife. One woman with two teen-aged daughters, while explaining her situation, said:

My husband stresses the righteous behavior of my two daughters. But they are studying in a German school, and though I can influence their behavior I cannot fully control them. … God forbid, if they do something wrong, my husband will never spare me. You know, “daughters’ behavior” is a matter of family prestige and honor. … And you know that people butcher their own daughters for the sake of their honor. I am afraid of that.

Other respondents also highlighted the growing problem of conflict and tension between parents and children**.** One woman told us that her teen-aged daughter was getting more and more annoyed and irritated with her because she didn’t like her (mother’s) controlling behavior. She further said: “My daughter distances herself from me [mother] because according to her I am nobody to control her dressing and movements.” Some women (9 of 32) expressed some fears such as *aolad-say-fasila* (intergenerational gap) as a cause of conflict and stress in families while living in Germany. Almost every respondent expressed a desire to preserve and transmit Pakistani values to their children. “If these values are under threat, we will simply go back; we cannot compromise”, said one mother of three small daughters. Another participant in her mid-thirties with a university degree pointed out: “Because of the language barrier, I cannot help my children in their school homework. My children think that I am a lay person, know nothing and they consider me backward. This is sometimes distressing for me.”

The third stressor reported by women was feelings of isolation and detachment from the host society. The women who participated in the study tended to have little formal education, which might have influenced their adjustment to the host society. Some women (6 of 32) with two to three years of migration history reported that they were living alone at home for the whole day. Few of them (5 of 32) felt estrangement from their neighborhood, geography, community, and perceived a low level of adjustment to the host society. These women reported a relatively restricted social life in Germany. Owing to various limitations, they usually interacted within the network of their own community. For years, they lived with their “own people” and rarely established contact with mainstream German society. One woman said: “Here people are nice but they are not very open or friendly. They are hesitant to make friendships and shy to contact foreigners.”

From the conversation it was revealed that a few women (4 of 32) had developed their own beliefs and stereotypes about mainstream German culture and had never tried to verify or check the validity of these beliefs. Based on these perceptions, they restricted their relations to “like-minded immigrant women”, especially from South Asian cultures. The majority of the women (19 of 32) reported that most of their time was spent on cooking and performing other household activities and they rarely went to libraries or recreational facilities or engaged in other activities. Nevertheless, almost half of the women in our study reported that they were quite satisfied with the freedom and sophisticated system of the host society.

Few women (4 of 32) felt very isolated and dissatisfied. Here, “isolation refers to the individual’s perception and reality of being emotionally and socially alone, economically confined, and culturally disconnected. It is the ‘feeling and fact’ of not belonging or having any meaningful relationship” (Abraham [1998], p. 224). Two women considered that this isolation was not natural; it was deliberately created by their families (husbands and in-laws) to keep them socially disconnected. One of the participants, while unfolding her memories, told us:

When I came to Germany for the first time, I was just 20 years old. I had many good dreams of living with my husband and going for outings. But when I came here the life was totally different. I had to live alone the whole day in my apartment. I waited for my husband the whole day. I felt totally insulted. I used to feel myself to be a bird in a cage.

One of the reasons for their isolation was the absence of extended family support for women. However, regarding the role of the extended family, the women reported contradictory situations. Some women (2 of 6) who had extended family in Germany considered the extended family itself to be a source of trouble for them. They thought that members of their extended family (e.g. mothers-in-law) tried to reinforce orthodox views about family and tried to make them into the “ideal good wife”. A majority of the women (22 of 32) thought that they were satisfied without their extended families (even though sometimes extended families played a supportive role). Overall, it was noted that immigrant families were trying to adopt and adjust to the host country’s realities with or without extended family*.*

### Women’s experiences of intimate partner violence (IPV) after immigration

#### Women’s attitude towards the problem of IPV in immigrant families

Dealing with IPV first requires an understanding of perceptions of and attitudes towards IPV by the population under study. In order to gain this insight, the participants were asked about their views on the problem of IPV as an issue in immigrant Pakistani families. Some of the women (8 of 32) opined that the problem of IPV against women is not a big issue in Pakistani immigrant families. They considered it to be a Western conspiracy and believed that the very concept of IPV was a “Western construct” and not a real problem to be worried about. “It is just propaganda of the Western media to defame Islam and Muslims”, commented one participant*.*

Another woman argued that Western culture had an “agenda” to disrupt Muslim family values. She believed that “mild and justified violence” is an essential component of marital stability. While presenting a “cost-benefit-analysis” of IPV she argued:

On the face of it, the Western family system seems very sober and civilized. But, in reality, the system is heading towards *tanazul* and *ikhlaqi dawaliapun* [social degeneration and moral bankruptcy]. Here women are free to go where they like. The father has no control over his daughter. The brother cannot ask his sister where and with whom she intends to spend nights. The net result is a growing number of divorces and couples living without marriages, without children.

Nevertheless, majority of the participants (24 of 32) did not seriously believe in conspiracy theories. Two high school educated housewives criticized the behavior of most of the poorly educated working-class men who exerted pressure on women to adjust their behavior according to the wishes of their husbands.

#### Women’s experiences of IPV

A growing body of research has shown that the incidence of IPV in immigrant communities is more or less similar to that in the host population but that the experiences of immigrant women in IPV situations are often aggravated by their specific positioning as immigrants (Menjivar and Salcido [2002]). Factors related to immigration, such as social isolation and lack of awareness about their rights or the existence of domestic violence services may place immigrant women at increased risk of IPV (Raj et al. [2005]).

We noted in our data that, because of their relatively low socioeconomic status, the immigrant families were deeply involved in solving different problems of adjustment, which also affected their spousal relations. Some women (9 of 32) reported that they had been facing many challenges, such as unemployment, housing, children’s schooling etc. after coming to Germany, but expressed their inability to help their husbands. While explaining her situation, one woman reported: “When my husband confronts a problem he gets angry; he gets irritated. After fixing the problem his behavior returns to normal. Sometimes, he apologies for his behavior. But for the time being, it is distressing for me.” Another respondent opined:

When we used to live in Pakistan, he sometime showed aggressive [violent] behavior. But there [in Pakistan] every situation was clear and predictable. Here, in Germany, things are different. Every day, we come across a new situation, a new problem. In many situations, he quickly loses his temper and starts abusing me. He blames me for many situations, though I am not at fault.

Most of the participants (18 of 32) reported that their relationships with their spouses became tense in “difficult situations” but that they returned to normal after that episode.

#### Psychological violence

Research has reported that among immigrant families psychological violence is common because of stressful situations (Thapa-Oli et al. [2009]). In this study we tried to explore the nature and extent of psychological violence among the immigrant families. The concept of psychological violence was first explained to the respondents. In this research we defined psychological violence as “consistently doing or saying things to shame, insult, ridicule, embarrass, demean, belittle, or mentally hurt another person” (Berry [1998], p. 3). In addition to this definition, certain situations were also described to the respondents in order to elaborate on the concept of psychological violence. Psychological violence was reported by most of the participants (21 of 32). Nonetheless, the women tried to blame the situation instead of their husbands. Only four women complained that they were “humiliated” by their husbands in various social situations and they directly accused their husbands of the deliberate use of psychological abuse. One thirty-year old participant with ten years of schooling narrated the following:

I am doing my best. I am doing what maximum I can do here to please my husband. But when my husband quarrels with me on some issue, he always says: “It would have been better if I had come here alone. It was my fault that I decided to bring you here.” And when I hear such things from his mouth, I feel very bad and humiliated.

One respondent, who was a single mother with an 11-year-old daughter and had been living in Germany for the last 17 years, told us:

Although I got divorced from my husband, I am still facing psychological violence from him. As my daughter spends every second weekend with him, he sometimes emotionally disturbs me on issues related to my daughter’s education and upbringing, which sometimes really makes me mad and emotionally unstable. He [ex-husband] knows my weaknesses.

One of the study participants described how the threat of violence was detrimental to her psychological and mental health. While narrating the impact of psychological violence on her personality, she stated: “I feel extreme shame when he threatens to slaps me. He doesn’t even care about the watching children. I can’t speak to my children because of the embarrassment.” However, three women reported a good change in their husbands’ behavior after moving to Germany. They reported that after coming to Germany, their husbands became more “civilized and caring” through the influence of German culture. However, overall, it was concluded that psychological violence was one of the most frequently committed abuses among the immigrant families than physical violence.

#### Physical violence

Physical violence against women occurs in all societies, although its manifestation, intensity and frequency varies and is linked with the level of economic development, and gender ideology (Menjivar and Salcido [2002]). In Pakistan, the most common forms of physical violence are pushing, slapping, punching, or hitting with an object (Fikree et al. [2005]).

For the present study, we asked the participants about the actual acts of physical violence they had ever experienced after migrating to Germany. However, before asking questions, we explained physical violence as “behaviors that threaten, attempt or actually inflict physical harm” (Crowell and Burgess [1996] p. 14). Six participants reported that they had experienced physical violence (in addition to psychological violence) after immigration. However, out of these, only one participant reported that she was subjected to serious acts of violence (hitting with a fist, kicking). Not even a single participant reported the use of some proscribed weapon by her husband, nor did they report any serious (life-threatening) injuries because of violence.

While discussing physical violence, women reported that in Germany men were deterred from committing open acts of physical abuse because of the strict laws and reasonably efficient police and judicial system. While commenting on women’s rights and the legal reaction to violence against women, one semi-literate participant observed:

Here in *pardes* [alien land, refereeing to Germany], men are careful about their hand [physical violence]. Here the police are very concerned about women’s rights. Women’s voices are quickly heard. Careless attitudes could cause immediate confinement of a violent man in jail. Our men know this.

### Women’s responses to intimate partner violence (IPV)

Women’s responses to IPV may be influenced by their immigration status as the new environment presents both new opportunities and challenges (Samuel [2009]). For example, their immigration status renders previous social skills and status irrelevant and also disrupts the social relations and family support system of the natal country (Sullivan et al. [2005]). Furthermore, women may not be able to seek help from the host country’s formal care-providing institutions because of the language barrier (Abraham [1998]; Bui [2003]; Yoshiama [2001]), and lack of knowledge about the legal system (Easteal [1996]) and community services (Weissman [2000]).

We asked questions about the respondents’ reactions and coping strategies in cases of IPV after coming to Germany. The data revealed that the women who were victims of different types of violence tried to resist or protect themselves from this violence by using different strategies to mitigate the tension and abuse and reconfigure their relationships with their husbands. The present study found that the abused women were in a difficult and complex situation because of the multiple uncertainties and insecurities they confront in the host country. One of the major difficulties reported by the women was the loss of their social network and the absence of support from their parental family members.

Most of the women who reported violence (12 of 21) were confused and undecided about adopting any particular strategy to resist violence. Actually the women tried to use multiple options to achieve the desired results. Almost all of the abused women wanted to use the “power of silence” to mend their husbands’ behavior. They hoped that the “circumstances will teach a lesson to the abusive husband.” They believed that the patience and wisdom of women should have enough power to “overpower a violent horse [husband].” The women also contacted their parental families back home for advice and support. This contact not only provided emotional solace to the women but their parents could also use their influence to put some moral pressure on the husband.

The data also showed that, with one exception, not a single woman wanted to seek help from the police or specialized care-providing services. Actually, these women did not want to increase their vulnerability; they were in no mood to take unnecessary risks in an alien land. The women who experienced abuse felt many kinds of vulnerabilities (these could be real or imaginary); they had a fear that if they complained to the police, then their husband may face deportation, which they did not want. Secondly, complaining about their husband may provoke him to take the extreme step of divorce and no woman wanted such an outcome.

The women who were still in abusive marriages were asked questions about their reasons for not leaving the abusive relationship. During the course of the interviews, it was noted that the women wanted to avoid the stigma of being a “problematic wife”. One woman said: “My life will be even worse if I get divorced.” Another woman observed: “In our culture divorce is not only the dissolution of the marriage, but it carries the stigma of women being ‘loose’, ‘immoral’ and ‘unlucky’.” Two women reported that, in the case of divorce, they would not be able to remarry and they would not be able to make an independent living. Going back to their parental home was wholly unacceptable for the women. It may be noted that bearing and suffering abuse in order to avoid the stigma of divorce was not only specific to Pakistani women. For example, women in India also tolerated IPV and they did not want to be as accused of “betrayal of their families” or to lose their identity as “Indian women” (Abraham [1998]; Mehrotra and Calasanti [2010]) and the combination of personal and family goals forced women to compromise and to stay in abusive relationships (Morash et al. [2008]).

Another reason to stay in an abusive relationship reported by some women was the primacy of their family institution. For these women, family was a part and parcel of their lives and they didn’t want to break up their families. The women knew that the damage caused by divorce would not be restricted to their personal life; the damage could be so pervasive and overwhelming that it could extend to the women’s parents and even to their siblings. The data revealed that the women wanted to avoid confrontation with their husbands. As a part of their socialization, most of them preferred to “wait for a better time”. In order to preserve their family honor, they wanted to avoid bickering and “kept it all inside the belly”. Women also know that their relatives and community will not encourage them if they air private family affairs in public. Another reason to remain in an abusive marriage was economic dependency on the husband.

Despite many limitations and vulnerabilities, Pakistani immigrant women were also aware of some “opportunities” and “positive points” about being in Germany. Firstly, they knew that their household work was being acknowledged and valued by their husbands. This may not be the case in Pakistan because of the joint or extended family system. For example, in Pakistan, somebody else (e.g., sister-in-law or mother-in-law) could perform a wife’s household work temporarily if she refuses or is unable to perform household routines. But in Germany, the women knew that nobody else could take over their responsibilities and, for that matter, their husbands were dependent on them.

#### Getting specialized care

Only one woman, who got divorced after bearing nine years of abuse from her husband in Germany, sought help from formal care-providing institutions. While narrating her perspective, she told that earlier she was afraid of the police. But when she contacted them, their behavior was different from Pakistani police. Based on her observations, she said that many South Asian women who experienced IPV did not know about the specialized services and institutions that are available to help such women. While narrating her own experiences in a *Frauenhaus* (German word for women’s shelter home) she stated:

For years, I had no knowledge about these services. One day, I visited the university with my ex-husband. There I saw a brochure from the social services office and then I called them for help. They were very cooperative. They arranged my stay in the Frauenhaus.

#### Obstacles to getting specialized care

During our interviews, the abused women narrated many factors which discouraged them from seeking help from formal institutions. The reasons include: (a) the inability to understand the structure and functioning of the care-providing institutions; (b) a lack of trust in the institutions’ capacity and commitment to solve the problems; (c) suspicion about the unknown and unpredictable consequences of the contact; and, (d) the women’s inability to explain their problem and convince people of their need for appropriate help.

## Discussion

The empirical data have shed light on the nature and dynamics of spousal relations in immigrant Pakistani families in Germany. The data revealed that, after immigration, couples faced various challenges in navigating through the different social, cultural, institutional, and legal environment of the host country. We noted that their immigration status posed various threats to immigrants’ self-identity, cultural values, and traditional gender roles.

Though our data supported the findings of earlier studies on immigrant women, they also challenged various stereotypes about immigrant Pakistani families, especially those relating to the attitudes of men towards women. First, not all women reported that their husbands had controlling or authoritative attitudes towards their wives. Second, after immigration, although men strived to protect their cultural values and identities, they also learnt from the host culture and became less abusive. Third, our data do not provide evidence to support the stereotype that immigrant women are passive victims of spousal abuse and wish to remain in social exclusion.

The women’s relatively social excluded role in German society was partly rooted in their pre-migratory gender socialization. This cultural upbringing restricted their capabilities to work within the household; they were not inclined to become engaged with the outside world. But despite this, the immigrant women were not reclusive or socially passive in the host society. Hence, immigrant women’s relatively restricted role in Germany may not be due to the controlling desire and “patriarchal mentality of the husband” but rather to women’s low level of education attained at their home country and the disadvantaged legal position offered to them by the host country (e.g., most of the women had dependent visa status and were not allowed to work).

In this research, we focused on IPV among immigrant Pakistani families. The present study showed that the prevalence of IPV among these immigrant women was relatively low compared to their counterparts in Pakistan. Though comparison is difficult because of methodological variations and present study’s tiny sample size, if we look at the prevalence of IPV in Pakistan, it is quite high; about 50% of married women in Pakistan are physically battered and 90% are emotionally and verbally abused by their husbands (Tinker [1998]). However, in our study, only a few (6 of 32) immigrant women reported physical violence while the majority of the women (21 of 32) reported psychological violence by their husbands. It could be interesting if these results are compared with IPV experienced by the women in the host country or other immigrant women living in Germany. Though comparison is difficult because of methodological variations and scarcity of IPV prevalence studies related to immigrant population, it is reported that 14% of German women and 28.6% women of Turkish origin experienced physical and/or sexual violence by their current partners in Germany (Condon and Schröttl [2006]). In another study (Ilkkaracan [1996]) nearly half of the Turkish immigrant women in Germany reported experiencing physical, psychological, financial or sexual violence from their husbands. It shows that Pakistani immigrant women in Germany were experiencing relatively less physical violence than Turkish immigrant women. Nonetheless, the prevalence of psychological violence may be the same.

In Pakistan, severe manifestations of patriarchy, such as physical beatings, honor killings and forced marriages are not uncommon, especially in rural areas (Johnson and Johnson [2001]). But no such event has been reported in our data. Rather, by coming to Germany, the nature of abuse has been changed, perhaps because of the different social and legal conditions in the host country.

The data revealed that immigration stressors influenced spousal relations but did not necessarily lead to spousal abuse. After immigration spousal relations may become tense, but women did not readily blame men, but the situation. One reason could be that women did not want to report abuse to avoid any unforeseen trouble. Rather they wanted to give their husbands the “benefit of the doubt” by considering that the abuse was due to situational factors created by immigration stressors and by hoping that “things will get better with the passage of time.” Leung and Cheung ([2008]) also found in the USA that Asian immigrants did not always perceive their partners’ abusive behavior as violence.

It was also noted that some of the women reported that their husbands had learnt from the experience and had become more polite and “civilized” through being exposed to German culture. The women were also aware of the fact that the relatively more efficient law-enforcement system in Germany was a deterrent to physical violence. So their immigrant status may not always be a “risk factor” for IPV but may also act as a “protective factor.” This may be similar in cases of women living without extended family after migration. Although living away from extended family may deprive women of valuable support and services (e.g. childcare), it may also render them vulnerable to abuse (Raj and Silverman [2002a]). At the same time, by living in a nuclear family, they may be safe from violence committed by extended family members. Further, extended family relations could also be instrumental in exacerbating spousal tension as they (extended family members) may encourage a husband to control his wife to “prove his masculinity” (Raj and Silverman [2002a]).

The data indicated a deep complexity in spousal relations among immigrant families as many “immigrant stressors” (e.g. unemployment, changed family relations, children’s socialization, the child-parent communication gap, social isolation, etc.) were contributing to increase spousal tension and conflict. But still a majority of the women thought that they had the capacity to overcome this difficult situation. As shown in the data, women tried various strategies to deal with the new situations. First, in the face of IPV, some women remained silent or passive. They actually used their silence as a strategy to “buy time.” Like other South Asian women, they considered tension and occasional conflict with their husbands to be a product of the unexpected situation.

Second, due to their socialization, they accepted the dominance of their husbands and were willing to tolerate the abuse, albeit for the time being. Such behavior is not only restricted to Pakistani women but Turkish immigrant women also used similar strategies to reduce tension and were successful in attaining some balance in their marital relations (Ilkkaracan [1996]). Third, the sanctity of the family was deeply ingrained in the minds of these women. That is why, with one exception, not even single women sought any professional help for IPV. Actually women do not want to “wash their dirty linen before others.” Asian immigrant women in the USA also exhibit similar behavior when they underutilize mental health services in order to hide their family problems (Leung and Cheung [2008]). It is the same with Arab women, who wanted to solve their spousal tension within the family instead of going to violence-prevention institutions (Haj-Yahia [2002]). Other studies on South Asian women have also reported that they remained silent about intimate relationships and conflicts (Abraham [1998]), partially because “they do not know they need not suffer abuse silently” (Paradkar [2000] p. M2).

The data showed mixed feelings of hope, fear, and a sense of vulnerability among immigrant Pakistani families in Germany. Women did not fully trust in the functioning of public institutions and remained shy about getting in contact with these institutions. Probably, the generally negative perceptions of the host country about immigrants in general, and Pakistanis in particular, may have instilled fear among the immigrant families. After 9/11, there has been a heightened sense of fear and insecurity among immigrants, not only in the USA but also in European countries. Recent research has reported that perceptions of threats (e.g. salience of the terrorism threat) can increase authoritarian tendencies in society (Fisher et al. [2010]). This sense of insecurity may infuse authoritarian tendencies into interpersonal relations such as parent–child and husband-wife. In such an environment, people may become more conservative, punitive and authoritarian when dealing with norm violators (Fisher et al. [2010]). Immigrant families, where men are already under identity threat (Kacen [2006]), may likely to persuade their wives to be more submissive and obedient.

### Limitations and strengths

As with all research, there were also some limitations to this study. One of the limitations of the present study was the nature of the sample and the study design. Because of difficulties in locating the target population, snowball sampling was used, which has its own limitations (e.g. sampling bias, sampling is inexact and non-representative, etc.). Second, the results documented by the study cannot be generalized to the overall population of Pakistani immigrants in Germany because the women who are more likely to be vulnerable to abuse might be reluctant to participate in the study. In our sample, the women were mostly from low-income working class families, so we cannot generalize these findings to the women from middle and high-income affluent families. Third, the sample was confined to three small cities in the North Rhine-Westphalia and Lower Saxony provinces of Germany. In these cities, Pakistani immigrant families are more sparsely scattered than in big German metropolitan areas such as Berlin or Frankfurt. Fourth, this study included only women’s perspective on the issue; the inclusion of men’s point of view could provide a relational perspective to the portrayal of Pakistani women living in Germany. Nevertheless, to the best of our knowledge, this study is the first exploratory study of immigrant women in Germany from Pakistan, which is a great strength of this study.

## Conclusion

This study addressed the issue of IPV against women and its related immigration stressors in Pakistani immigrant families in Germany. The data indicated a deep complexity in spousal relations among immigrant families as many “immigrant stressors” (e.g. unemployment, changed family relations, children’s socialization, the child-parent communication gap, social isolation, etc.) were contributing to increase spousal tension and conflict. But still a majority of the women thought that they had the capacity to overcome this difficult situation. This study debunks some stereotypes and popular media clichés (e.g., women are passive victims and socially disintegrated) about the “victimhood of women from conservative developing countries.” So, this research can meaningfully contribute to the understanding of this complex issue within an immigration context. Further qualitative research with both men and women could continue to explore the phenomenology and meanings attributed to IPV by the immigrant women. It is also important to further investigate the pathways and social mechanism of various immigration stressors such as social exclusion, distancing, work-place exploitation and their linkages to IPV.

## Abbreviations

IPV, Intimate partner violence; TV, Television.

## Competing interests

The authors have no competing interests.

## Authors’ contributions

RZ conceived the idea and carried out the study, collected data, and drafted the manuscript. MZZ contributed in framing the study design, and provided input in interpretation of data and in drafting the manuscript. TF and AK revised the manuscript and contributed to improve its readability, intellectual contents and scientific objectivity. All authors read and approved the final manuscript.
